# Characteristics and Outcomes of External Referral Patients Undergoing Cytoreductive Surgery at an International Peritoneal Surface Malignancy Program

**DOI:** 10.1245/s10434-026-20115-3

**Published:** 2026-07-18

**Authors:** Edward A. Joseph, Muhammad Muntazir Mehdi Khan, Catherine Lewis, David L. Bartlett, Patrick L. Wagner, Casey J. Allen

**Affiliations:** 1 Allegheny Health Network Singer Research Institute, Pittsburgh, PA USA; 2 Division of Surgical Oncology, Department of Surgery, Allegheny Health Network, Pittsburgh, PA USA; 3 Cancer Institute, Allegheny Health Network, Pittsburgh, PA USA

**Keywords:** Peritoneal carcinomatosis, Hyperthermic intraperitoneal chemotherapy, Completeness of cytoreduction

## Abstract

**Background:**

Peritoneal surface malignancy (PSM) patients often seek care outside their local health systems. Understanding the implications of care for these externally referred patients is critical for optimizing resource allocation and value-based care delivery.

**Patients and Methods:**

Patients who underwent cytoreductive surgery ± heated intraperitoneal chemotherapy (CRS ± HIPEC) at an international PSM program between 2022 and 2024 were categorized as in-system (IS; referrals within our integrated network) or out-of-system (OOS; referrals from external institutions). Demographic, oncologic, surgical, and financial data were compared.

**Results:**

Among 171 patients (mean age: 56 ± 12 years; 57% female; 92% white), 59% (*n* = 101) were OOS. These patients had higher Peritoneal Carcinomatosis index (PCI) (18.5 [12.0–29.0] versus 13.0 [6.0–18.0]), longer operative time (518 [381–630] min versus 321 [214–490] min), and hospitalizations (14.0 [9.0–21.0] days versus 9.0 [6.0–15.0] days), all *p* < 0.050. There was no difference in completeness of cytoreduction (CC 0/1: 85.0% OOS versus 87.7% IS, *p* = 0.626), readmissions (20.8% OOS versus 15.7% IS, *p* = 0.403), or mortality (4.0% OOS versus 0.0% IS, *p* = 0.092). OOS patients incurred higher institutional costs (direct: $76,620 [$56,527–$99,072] versus $58,375 [$35,363–$82,635], *p* < 0.001; indirect: ($30,788 [$20,581–$40,037] versus $20,743 [$12,690–$35,887], *p* < 0.001). Notably, of 72 (71%) OOS patients previously deemed unresectable at outside institutions, 85% (*n* = 61) achieved complete and 10% (*n* = 7) received palliative cytoreduction.

**Conclusions:**

OOS patients with PSM present with higher disease burden, require more extensive surgery, and incur higher costs. Yet, most OOS patients previously deemed unresectable were able to achieve complete cytoreduction at a high-volume PSM program.

**Supplementary Information:**

The online version contains supplementary material available at https://doi.org/10.1245/s10434-026-20115-3.

## Background

Peritoneal surface malignancy (PSM) commonly arises from advanced gastrointestinal and gynecological malignancies and is characterized by intraabdominal spread, significantly impacting overall survival.^1,2^ Over the past three decades, growing insights into the tumor dissemination pathways within the peritoneal cavity have reframed PSM as a loco-regional disease rather than a systemic process.^3^ Consequently, aggressive, multimodal approaches, combining cytoreductive surgery (CRS) and hyperthermic intraperitoneal chemotherapy (HIPEC), have emerged as promising strategies to enhance regional control and improve survival outcomes among patients with PSM.^4–7^ In recent years, the number of centers performing CRS ± HIPEC in the USA has steadily increased, expanding access to this specialized treatment.^8^ Accompanying this expansion, there is a rising proportion of patients traveling from outside their local networks to receive specialized care at high-functioning facilities.^9,10^ However, as the success of CRS ± HIPEC depends on several factors,^11–13^ despite the rising number of external referrals, studies have not directly explored the characteristics and outcomes of this specific patient population, both in terms of how these patients’ disease differ from those treated within their local networks and the resource implications of what caring for these patients might bring to a single institution. Investigating these differences will help us understand what is required to optimize care delivery to patients with PSM in the USA. This study aims to characterize externally referred patients undergoing CRS ± HIPEC at a quaternary PSM program. By understanding their disease, treatment outcomes, and patterns of resource utilization, we aim to better inform strategies to optimize their care.

## Patients and Methods

This study was approved by the Allegheny Health Network Institutional Review Board on 13 May 2022 (protocol # 2022-078-AHNMR).

### Study Population, Data Acquisition, and Variables of Interest

This retrospective, comparative analysis included patients who underwent CRS ± HIPEC between January 2022 and March 2024 within a large integrated cancer network program. This institutional database utilized for this study consisted solely of patients who ultimately underwent operative management and was constructed by integrating patient electronic medical records, which provided demographic information, admission and discharge dates, and diagnoses, along with detailed surgical, oncological, and cost data from various institutional sources. Surgical and perioperative information, including Peritoneal Carcinomatosis index (PCI) scores, completeness of cytoreduction (CC) scores, length of stay (LOS), operative time, estimated blood loss, Clavien–Dindo complication grade, readmission rate, and 90-day mortality, were abstracted from operative and anesthesia notes, while histological diagnoses were confirmed through pathology records. Institutional costs were obtained from our health network’s finance department, and 90-day direct and indirect institutional costs were compared.

### Cohort Stratification

Patients were divided into two distinct cohorts: in-system (IS), consisting of patients who were referred from within our regional health network (a 14-hospital health system centered in Pittsburgh, PA, which serves 29 counties in Pennsylvania and portions of New York, Ohio, and West Virginia),^14^ and out-of-system (OOS), consisting of patients who were referred to us from outside our health system. For OOS patients, we obtained health records to review prior care received at outside facilities, including whether they were offered cytoreduction and their surgical outcomes when appropriate.

### Cost Analysis

Direct institutional costs were defined as direct variable costs and direct fixed costs. Direct variable costs consisted of three categories: direct variable salary, direct variable supply, and direct variable other. Direct variable salary included salaries and benefits from direct patient care departments. Direct variable supply included all patient care supply expenses from direct patient care departments. Direct variable other included all other accounts that were not a supply or salary account within a direct patient care cost center. Direct fixed costs consisted of two categories: direct fixed salary and direct fixed other. Direct fixed salary included salaries and benefits from direct patient care departments. Direct fixed other included all other accounts that were not a supply or salary account within a direct patient care cost center. Indirect institutional costs were defined as both indirect salary and indirect other expenses, including utilities, interest, depreciation, amortization, and taxes. These are costs that are reported in the budget execution (BEX) reports of cost centers that do not provide direct patient care and are identified as non-patient care cost centers. Examples include security, maintenance, medical records, administration, and so on. We compared 90-day direct and indirect institutional costs between the groups.

### Statistical Analysis

Parametric data are reported as mean ± standard deviation, while nonparametric data are reported as median and interquartile range. Basic descriptive statistics were employed to analyze demographic and cancer-specific data, including means and standard deviations for continuous variables and proportions and frequencies for categorical variables. To evaluate differences between IS and OOS patients who underwent CRS ± HIPEC at our facility, the Student’s *t*-test was employed for parametric data, and the Mann–Whitney *U* test was applied to nonparametric data. Categorical data were compared using the Pearson chi-squared test; when cell counts were lower than 5, the Fisher’s exact test was utilized. Pearson correlation tests were conducted to assess associations between continuous variables. Significance was established at a *p*-value of less than 0.050. All statistical analyses were performed using SPSS Version 28 (IBM Corporation, Armonk, NY) and STATA version 19.

As a sensitivity analysis, the interval between the approximate referral date from the outside institution and surgery date at our center was evaluated among the OOS patients to assess whether referral timing influenced disease burden or perioperative outcomes. Referral date was selected rather than date of diagnosis given the prolonged heterogenous disease trajectories common among patients with PSM. Patients were stratified into quartiles on the basis of their referral-to-surgery interval and compared with respect to PCI, completeness of cytoreduction, operative characteristics, and postoperative outcomes.

## Results

Our cohort included 171 patients, of which 70 were IS patients and 101 were OOS patients. They were 55.8 ± 12.3 years old, 91.8% (*n* = 157) white, and 56.7% (*n* = 97) female. Histologically, the most common diagnoses included adenocarcinoma of the appendix (32.2%; *n* = 55), adenocarcinoma of the colon (22.2%; *n* = 38), low-grade appendiceal mucinous neoplasm and high-grade appendiceal mucinous neoplasm (22.2%%;* n* = 38), carcinoma of the ovary (9.9%; *n* = 17), mixed peritoneal mesothelioma (3.5%; *n* = 6), mixed peritoneal sarcomatosis (2.3%; *n* = 4), adenocarcinoma of the small intestines (1.2%; *n* = 2), high-grade Müllerian carcinoma (1.2%; *n* = 2), and other histologic diagnoses including, metastatic lobular carcinoma of the breast (1.2%; *n* = 2), serous cystadenoma (1.2%; *n* = 2), metastatic renal cell carcinoma (0.6%, *n* = 1), metastatic perivascular epithelioid cell tumor (PEComa) (0.6%, *n* = 1), metastatic gastric adenocarcinoma (0.6%, *n* = 1), and metastatic urachal carcinoma (0.6%, *n* = 1). For the overall cohort, median PCI was 15.0 (9.0–26.0), and most patients achieved a complete/near-complete cytoreduction (CC0/1: 83.0%; *n* = 142) at our institution, with a median length of stay of 11.0 (8.0–19.0) days (Table 1).

**Table 1:** Clinicodemographic characteristics of the overall cohort

N	171 (100.0%)
Age^a^	55.8 ± 12.3
*Sex*	
Male	74 (43.3%)
Female	97 (56.7%)
*Race*	
White	157 (91.8%)
Black	3 (1.8%)
Asian	5 (2.9%)
Hispanic	2 (1.2%)
Other	4 (2.3%)
*Insurance status*	
Private	115 (67.3%)
Government	42 (24.6%)
Self-pay	12 (7.0%)
Other	2 (1.1%)
*Histology*	
LAMN/HAMN	38 (22.2%)
Appendiceal adenocarcinoma	
Well-to-moderately differentiated	26 (15.2%)
Poorly differentiated	29 (17.0%)
Colon adenocarcinoma	
Well-to-moderately differentiated	22 (12.8%)
Poorly differentiated	16 (9.4%)
Ovarian carcinoma	
Low grade serous	10 (5.8%)
High grade serous	3 (1.8%)
Borderline mucinous	4 (2.3%)
Small bowel adenocarcinoma	2 (1.2%)
Mesothelioma	6 (3.5%)
Sarcoma	4 (2.3%)
High grade mullerian carcinoma	2 (1.2%)
Other/Miscellaneous	9 (5.2%)
*Prior chemotherapy*	89 (52.0%)
*PCI*^b^	15.0 (9.0–26.0)
*PCI ranges*	
< 10	24 (21.1%)
10–20	39 (34.2%)
21–30	26 (22.8%)
> 30	25 (21.9%)
*CC score at ur facility*	
CC0/1	142 (83.0%)
CC2/3	29 (17.0%)
*LOS*^b^	11.0 (8.0–19.0)

^a^Data reported as mean ± standard deviation, ^b^data reported as median and interquartile range; LAMN, Low-grade appendiceal mucinous neoplasm; HAMN, High-grade appendiceal mucinous neoplasm; PCI, Peritoneal Carcinomatosis index; CC, Completeness of cytoreduction; LOS, Length of stay

**Fig. 1:**
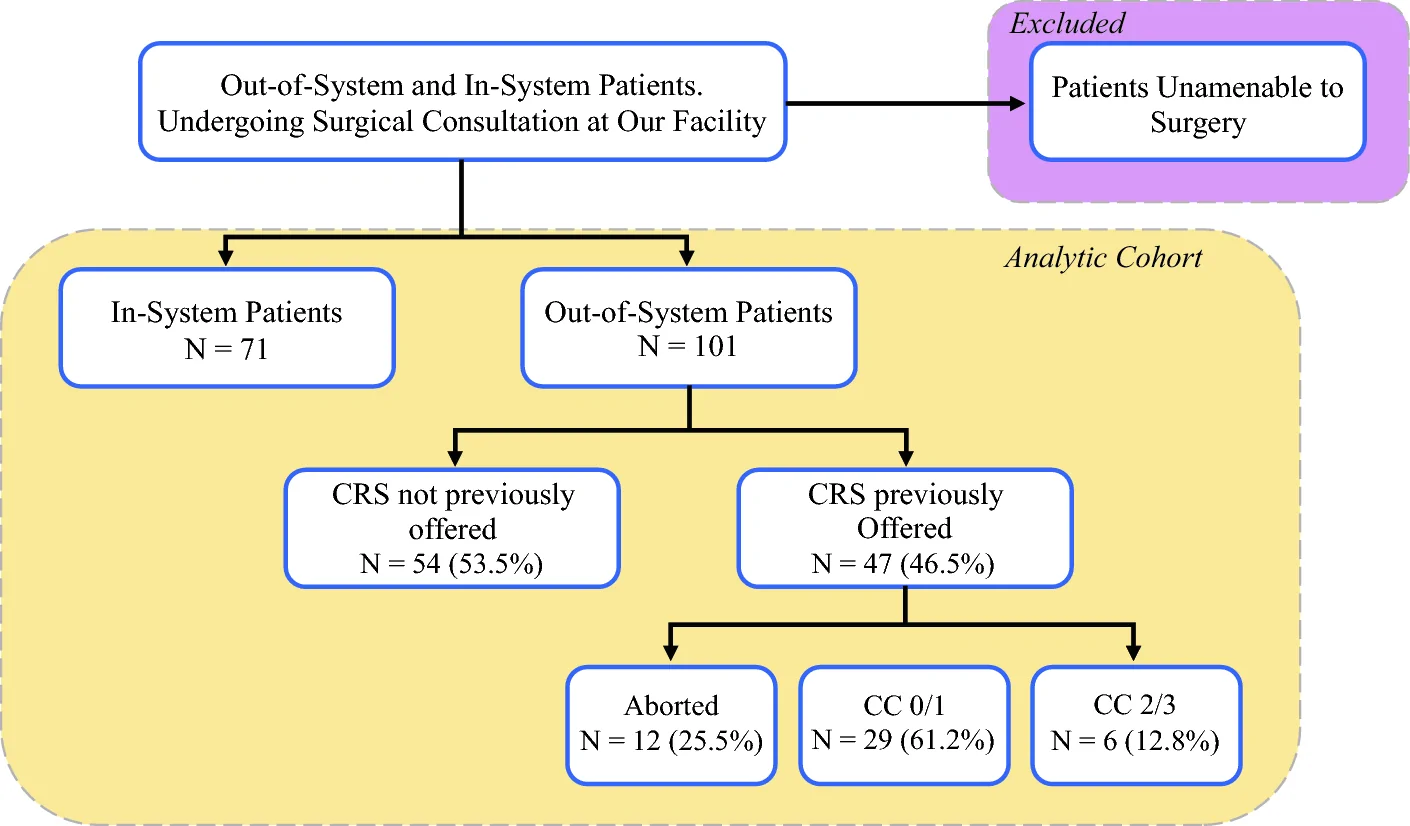
Flowchart describing the analytical cohort and prior management and cytoreductive outcomes of out-of-system (*n* = 101) patients before referral to a quaternary peritoneal surface malignancy program; CRS, Cytoreductive surgery; CC, Completeness of cytoreduction

The OOS patients were younger (53.8 ± 12.0 OOS versus 58.9 ± 12.2 IS, *p* = 0.007) and less likely to be female (female: 50.5% OOS versus 65.7% IS, *p* = 0.048), but there was no difference in race (white: 92.1% OOS versus 94.1% IS, *p* = 0.859) between the two cohorts. In patients with appendiceal cancer, there was a higher proportion of OOS patients who presented with poorly differentiated histology (22.8% OOS versus 8.6% IS, *p* = 0.001) (Table 2). Repeat cytoreduction procedures were included in both cohorts, with 45.1% of patients having undergone at least one prior cytoreduction before the index operation (Supplementary Table 1). Externally referred patients originated from a broad geographic area spanning multiple regions of the USA and international referral pathways (Supplementary Fig. 1). Of all OOS patients, 53.5% (*n* = 54) were not previously offered CRS at an outside facility. Of the OOS patients who did undergo a prior attempt at CRS at an outside facility (46.5%; *n* = 47), 25.5% (*n* = 12) were aborted intraoperatively, and 12.8% (*n* = 6) received an incomplete cytoreduction (CC ≥ 2) (Fig. 1). All of the OOS patients received evaluation at a cancer center and the majority (85.9%) were referred by board certified complex general surgical oncologists (Supplementary Table 2).

**Table 2:** Comparison of clinicodemographic characteristics between in-system (*n* = 70) and out-of-system (*n* = 101) patients who underwent cytoreductive surgery ± heated intraperitoneal chemotherapy at a quaternary peritoneal surface malignancy program

	In-system (*n* = 70)	Out-of-system (*n* = 101)	*p*-value
*Age*^a^	58.9 ± 12.2	53.8 ± 12.0	0.007
*Sex*			0.048
Male	24 (34.3%)	50 (49.5%)	
Female	46 (65.7%)	51 (50.5%)	
*Race*			0.859
White	64 (91.4%)	93 (92.1%)	
Black	2 (2.9%)	1 (1.0%)	
Asian	2 (2.9%)	3 (3.0%)	
Hispanic	1 (1.4%)	1 (1.0%)	
Other	1 (1.4%)	3 (3.0%)	
*Insurance status*			0.057
Private	48 (68.6%)	67 (66.3%)	
Government	20 (28.6%)	22 (21.8%)	
Self-pay	1 (1.4%)	11 (10.9%)	
Other	1(1.4%)	1 (1.0%)	
*Histology*			0.001
LAMN/HAMN	12 (17.1%)	26 (25.7%)	
Appendiceal adenocarcinoma			
Well-to-moderately differentiated	7 (10.0%)	19 (18.8%)	
Poorly differentiated	6 (8.6%)	23 (22.8%)	
Colon adenocarcinoma			
Well-to-moderately differentiated	9 (12.8%)	13 (12.9%)	
Poorly differentiated	6 (8.6%)	10 (9.9%)	
Ovarian carcinoma			
Low grade serous	9 (12.9%)	1 (1.0%)	
High grade serous	3 (4.3%)	0 (0.0%)	
Borderline mucinous	3 (4.3%)	1 (1.0%)	
Small bowel adenocarcinoma	1 (1.4%)	1 (1.0%)	
Mesothelioma	4 (5.7%)	2 (2.0%)	
Sarcoma	3 (4.3%)	1 (1.0%)	
High grade mullerian carcinoma	2 (2.9%)	0 (0.0%)	
Other/miscellaneous	5 (7.1%)	4 (3.9%)	
*Prior chemotherapy*	39 (55.7%)	50 (49.5%)	0.272
*PCI*^b^	13.0 (6.0–18.0)	18.5 (12.0–29.0)	< 0.001
*CC score at our facility*			
CC0/1	57 (87.7%)	85 (85.0%)	0.626
LOS^**b**^	9.0 (6.0–15.0)	14.0 (9.0–21.0)	< 0.001

^a^Data reported as mean ± standard deviation, ^b^data reported as median and interquartile range; LAMN, Low-grade appendiceal mucinous neoplasm; HAMN, High-grade appendiceal mucinous neoplasm; PCI, Peritoneal Carcinomatosis index; CC, Completeness of cytoreduction; LOS, Length of stay

**Fig. 2:**
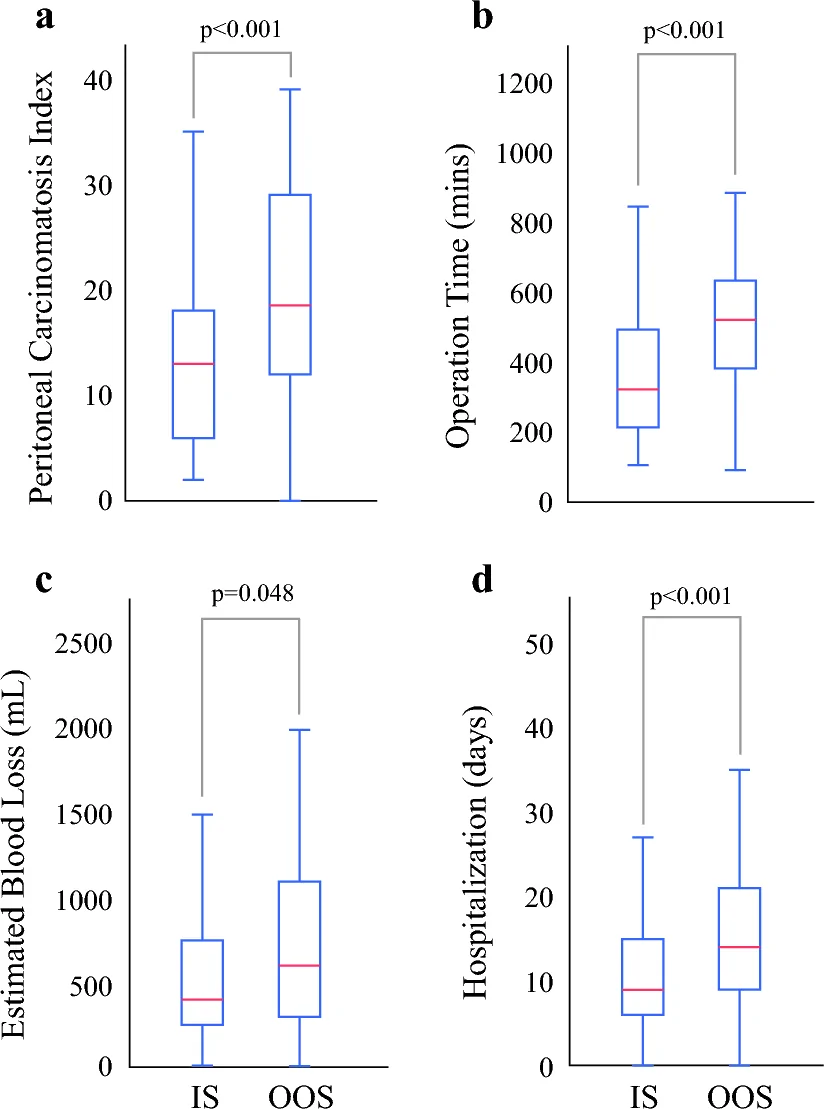
Box-and-whisker plots comparing perioperative outcomes in-system (*n* = 70) and out-of-system (*n* = 101) patients following cytoreductive surgery at a quaternary peritoneal surface malignancy program, (**a**) Peritoneal Carcinomatosis index (PCI): OOS patients presented with significantly higher PCI scores, (**b**) operation time: OOS patients had longer operative durations, (**c**) estimated blood loss: OOS patients experienced greater intraoperative blood loss, (**d**) hospitalization length: OOS patients had longer postoperative hospital stays; boxes represent the interquartile range, horizontal lines indicate the median, and whiskers denote the range; *p* < 0.050 was considered statistically significant

**Fig. 3:**
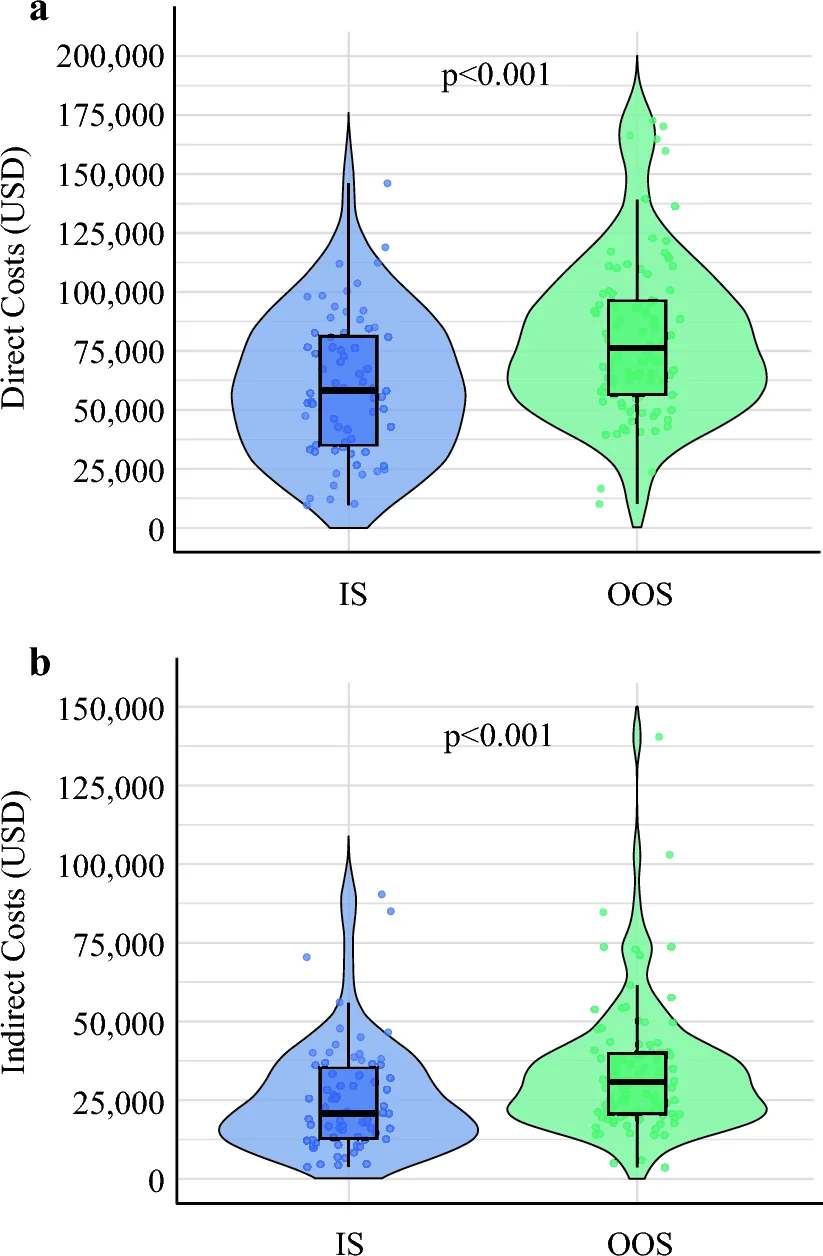
Violin charts comparing the 90-day direct and indirect institutional costs in-system (*n* = 70) and out-of-system (*n* = 101) patients following cytoreductive surgery at a quaternary peritoneal surface malignancy program, (**a**) 90-day direct institutional costs, (**b**) 90-day indirect institutional costs; each violin displays the full distribution of cost data, with internal boxplots indicating the interquartile range and horizontal lines denoting the median; *p* < 0.050 was considered statistically significant

Subsequent to referral, there was no difference in preoperative systemic chemotherapy between the two cohorts (49.5% OOS versus 55.7% IS, *p* = 0.272), OOS patients had higher PCI (18.5 [12.0–29.0] versus 13.0 [6.0–18.0], *p* < 0.001), longer operative time (518 [381–630] min versus 321 [214–490] min, *p* < 0.001), higher estimated blood loss (600 [300–1100] mL versus 400 [250 – 800] mL, *p* = 0.048), and longer hospitalizations (14.0 [9.0–21.0] days versus 9.0 [6.0–15.0] days, *p* < 0.001) (Fig. 2). Of the two groups, The OOS cohort underwent more extensive cytoreductive procedures, including higher rates of left diaphragm resection, cholecystectomy, small bowel resection, low anterior resection, and ostomy creation (all *p* < 0.005). Detailed procedure specific comparisons can be found in Supplementary Table 3. Furthermore, OOS patients had a higher frequency of HIPEC utilization in comparison with the IS cohort (73.3% versus 31.4%, *p* < 0.001) (Supplementary Table 4). However, there was no difference in the CC scores (CC 0/1: 85.0% OOS versus 87.7% IS, *p* = 0.626), Clavien–Dindo complications (grade ≥ 3: 36.6% OOS versus 28.6% IS, *p* = 0.271), and readmission rates (20.8% OOS versus 15.7% IS, *p* = 0.403). Moreover, there was no difference in 90-day mortality (4.0% OOS versus 0.0% IS, *p* = 0.092) between the two groups. The OOS patient cohort accrued higher 90-day direct ($76,620 [$56,527–99,072] versus $58,375 [$35,363–82,635],* p* < 0.001) and indirect ($30,788 [$20,581–40,037] versus $20,743 [$12,690–35,887], *p* < 0.001) institutional costs (Fig. 3). There was no difference in overall survival between the two groups (*p* = 0.135) (Supplementary Fig. 2).

On sensitivity analysis restricted to OOS patients, the referral-to-surgery interval was stratified into quartiles to evaluate whether timing of referral was associated with disease burden or operative outcomes. No differences were observed across quartiles with respect to PCI (*p* = 0.248), PCI category (*p* = 0.213), completeness of cytoreduction (*p* = 181), grouped CC status (*p* = 0.749), estimated blood loss operative time (*p* = 0.102), length of stay (*p* = 0.844), or prior chemotherapy exposure (*p* = 0.299) (Supplementary Table 5).

Of all OOS patients, 71% (*n* = 72) were unable to receive complete cytoreductions at outside facilities (54 not offered surgery, 12 were aborted intraoperatively, 6 underwent incomplete cytoreduction). Of these, the majority subsequently achieved complete cytoreduction (85%, *n* = 61) or surgical palliation (10%, *n* = 7) at our PSM center.

## Discussion

In this study, we found that externally referred patients with PSM represent a distinct patient population with a more aggressive disease burden, incurring greater treatment complexity and resource utilization compared with patients referred from within our own health system. Still, the majority of these patients were able to receive oncologically beneficial CRS and/or palliation.

PSM remains among the most challenging oncologic conditions, arising from advanced gastrointestinal and gynecologic cancers and often associated with diffuse intraabdominal spread.^15,16^ Once considered a terminal state with few effective options, outcomes have been redefined by advanced management strategies such as CRS ± HIPEC.^17–19^ These approaches have been shown to improve survival in selected patients, yet its success depends significantly on achieving complete cytoreduction and underlying tumor biology.^20,21^ However, delivering CRS ± HIPEC effectively also requires considerable institutional capacity, including specialized perioperative resources and experienced surgical teams.^22,23^ Formal PSM programs might bring these elements together, enabling safe execution of complex cytoreductions while minimizing morbidity.^23^ In this setting, patients with cases that might otherwise be considered unresectable can often undergo meaningful interventions, highlighting the central role of specialized centers in expanding the therapeutic potential of CRS ± HIPEC for patients with PSM.

One of the central observations of this study is that externally referred patients represent a distinct subset of the PSM population. Compared with IS patients, OOS patients had higher PCI scores and a greater prevalence of poorly differentiated histologies. The greater disease burden observed among OOS patients is likely the result of a referral bias, wherein patients with more advanced disease were directed to specialized centers for management, but it may also be suggestive of inefficiencies in referral timing and care coordination.^9,24^ Similar to the result of our study, in a prior analysis by Solsky et al., patients who traveled further distances had higher PCI scores, raising questions about how delayed referrals or a lack of multidisciplinary evaluation can contribute to disease progression before surgical assessment.^9^ PSM are complex to manage and require early consultation within a multidisciplinary team to identify candidates for CRS ± HIPEC.^16^ Patients with PSM are frequently managed with systemic chemotherapy as a part of multimodal treatment strategies prior to referral for surgical evaluation, although prolonged nonoperative management may in some cases contribute to delayed evaluation for CRS ± HIPEC and potentially narrow the optimal window for cytoreduction.^25^ Importantly, all of the patients within the OOS cohort had a documented prior evaluation at a cancer center, with the majority being referred by surgical oncologists. This suggests that referral to specialized PSM centers is a reflection of the operative complexity and institutional demand associated with patients who have advanced peritoneal disease rather than the absence of effective oncologic evaluation alone. Accordingly, these findings delineate that while many patients undergo appropriate initial oncologic evaluation within established cancer centers, select patients with advanced PSM may ultimately benefit from evaluation at a specialized PSM referral center equipped to manage the technical and perioperative demands of extensive cytoreduction. That being said, although prior studies have suggested that that delayed or fragmented referral processes may contribute to more extensive disease at presentation, our sensitivity analysis of the OOS patients did not demonstrate an association between referral interval and disease burden or operative outcomes.^25^ As such, the greater disease burden observed within this cohort likely reflects a more complex interplay of referral patterns, disease biology, and institutional selection patterns.

In comparison with the IS patients, externally referred patients experienced longer operative times, higher intraoperative blood loss, and extended hospitalizations, yet the majority ultimately achieved complete or near complete cytoreductions. The perioperative findings within our study further elaborate on those from a national study by Sadjadi et al. evaluating patients who traveled long distances to receive CRS ± HIPEC.^10^ The consistent pattern of differences in perioperative outcomes observed corroborate our understanding that the externally referred patient population present with more extensive disease and require greater operative and institutional resources. This understanding is further supported by a Canadian feasibility study that described patients with advanced disease traveling to undergo definitive cytoreduction at specialized centers.^24^ Collectively, our findings suggest that referral status is not merely an administrative distinction but is instead representative of disease severity, operational complexity, institutional demand, and the prior care pathways leading to surgery for this cohort of patients.

Interestingly, our analysis revealed that a significant proportion of externally referred patients had been previously deemed unresectable or experienced incomplete or aborted surgery at outside facilities. Several factors may contribute to these outcomes. Prior literature suggests that a higher burden of poorly differentiated or high-grade histology is associated with a lower likelihood of achieving complete cytoreduction.^25,26^ In an earlier study, around 44.0% of aborted cases harbored poorly differentiated tumors compared with only 22% in those successfully resected, emphasizing the biological aggressiveness of these presentations.^25^Authors have identified a number of factors including the extent of disease, anatomic distribution, histologic subtype, and the technical feasibility of achieving macroscopic clearance as major determinants of intraoperatively aborted cytoreductions.^25,27^ Among these, the PCI remains the most prognostic factor for oncologically beneficial cytoreduction.^28,29^ Multiple investigators have argued for prognostic PCI thresholds, with cutoffs ranging from 13 to 20 serving as important delineators of survival, beyond which many institutions exercise greater caution in pursuing cytoreduction.^29,30^ Still, PCI alone might not preclude beneficial cytoreduction, as long-term survival remains achievable even in patients with extensive disease when treated at experienced, high-volume centers.^31^ This reinforces the pivotal role of specialized centers and multidisciplinary evaluation to safely manage patients with advanced disease who might otherwise be denied surgery elsewhere.

Beyond the clinical complexity of this cohort, an equally important consideration concerns the substantial investment required to care for externally referred patients with PSM. The higher disease burden associated with externally referred patients naturally translates into the need for greater operative time, support, and institutional investment. However, these costs must be interpreted in the context of value-based care, wherein the central tenet of care delivery focuses not simply on cost containment, but rather also on aligning resource utilization with meaningful patient outcomes.^32–36^ In this regard, the additional resources needed for the externally referred patient cohort translate to significant survival benefits. In a survey-based assessment of quality of life (QOL) of patients who underwent CRS ± HIPEC, Knotts et al. demonstrated that patients consistently ranked survival as the most important care priority, far exceeding concerns regarding the costs of care and potential perioperative morbidity.^34^ These findings underscore that, for appropriately selected patients, the perceived value of undergoing an intensive, resource-heavy procedure is inherently tied to the possibility of durable disease control and extended survival. From an institutional standpoint, the higher complexity of these cases demands multidisciplinary coordination, experienced surgical teams, and robust perioperative infrastructure, all of which will incur substantial expenditure but are essential to achieve optimal outcomes for patients with higher disease burden. Therefore, the true measure of value in specialized CRS ± HIPEC centers lies in effectively balancing costs with oncologic efficacy, recognizing that the pursuit of high-quality, equitable, and robust outcomes often requires proportional investment and judicious resource allocation.

As the number of centers offering CRS ± HIPEC in the USA and internationally continues to grow,^8,37^ understanding how to effectively manage externally referred patients becomes essential for maintaining quality and efficiency of care delivery. Our study provides a useful benchmark for understanding this evolving referral landscape, defining the clinical, operative, and institutional attributes of externally referred patients who typically present with higher burden of disease, more aggressive biology, and greater perioperative complexity. Despite these challenges, comparable outcomes can be achieved through the development of coordinated referral networks and the consolidation of complex cases at high-functioning, specialized PSM centers equipped with the multidisciplinary expertise and perioperative infrastructure required for such demanding cases. This notion is supported by data from the Netherlands, which demonstrated a clear-cut improvement in outcomes and survival following the implementation of structured referral networks, where disparities in access to CRS ± HIPEC and overall survival between referring facilities and HIPEC centers were effectively eliminated, underscoring the system-level impact of coordinated referral pathways.^38^ Building on such models, future efforts should emphasize early identification of surgical candidates, cross-institutional education, and shared decision-making frameworks to promote timely referrals and broaden institutional capacity. By integrating these principles, developing CRS ± HIPEC programs can anticipate the clinical and logistical needs of externally referred patients, optimize resource allocation, and ensure that the expansion of CRS ± HIPEC availability is accompanied by a consistent standard of oncologic excellence.

This study has certain limitations to consider. Its retrospective, single-center design introduces the potential for both selection and referral bias, as patients reaching a high-volume quaternary PSM referral center likely represent a highly selected cohort of complex advanced or previously unresectable cases. Referral patterns may additionally reflect institutional reputation, established multidisciplinary expertise, existing referral networks, and patient-driven care-seeking behaviors. Consequently, the operative outcomes and care pathways observed within this study may not be directly reproducible at lower-volume institutions or centers without comparable perioperative infrastructure and CRS ± HIPEC experience, limiting the generalizability of our findings. Nevertheless, these characteristics reflect the real-world function of specialized referral centers and provide insight into the unique clinical and operational demands associated with managing externally referred patients with PSM. The institutional dataset used for this study was constructed from patients who ultimately underwent CRS ± HIPEC and therefore did not systematically capture patients referred for surgical evaluation but managed nonoperatively. Consequently, the study is unable to characterize the full referral denominator or institutional selection patterns, introducing another potential avenue for referral and operative selection bias. Despite all externally referred patients having documentation of prior consultation at a cancer center, patients frequently visited multiple hospitals prior to their consultation at our facility, and the detailed institutional characteristics of these facilities were not consistently available. Consequently, the study was unable to reliably characterize the referring institutions’ facility type, operative volume, CRS ± HIPEC-specific experience, or surgeon-level expertise. These factors may influence referral patterns, operative decision-making, and generalizability of the findings. Although the study integrates detailed clinical, operative, and financial data, cost analyses were based on institutional accounting frameworks and may not be directly comparable across centers or payer models. Moreover, indirect and patient-borne costs, such as travel, lodging, and loss of income, were not captured, underestimating the broader economic burden of care. The study was also limited by its lack of quality-of-life outcomes. In addition, the heterogeneity of tumor biology and histologic subtypes may have introduced residual confounding despite cohort stratification. Although receipt of preoperative systemic therapy was captured, granular details regarding the number of prior lines and duration of treatment were not consistently available from outside records and therefore could not be reliably analyzed. Although survival analyses were performed, the study was not designed or powered to detect histology-specific differences in long-term oncologic outcomes, and follow-up duration may be insufficient to fully characterize survival among patients undergoing CRS ± HIPEC. Despite having patients’ geographical and insurance information available, granular socioeconomic variables including income, education, and area-level deprivation indices were not captured. Future prospective, multi-institutional studies with standardized data collection, longer follow-up, and standardized cost reporting are needed to validate these findings and better characterize the outcomes and economic implications of externally referred patients undergoing CRS ± HIPEC.

## Conclusions

Patients with PSM referred from outside their regional health system for CRS ± HIPEC represent a distinct and clinically important subgroup. They present with more aggressive disease, necessitating greater operative complexity and institutional resource utilization. Nevertheless, most of these patients may achieve meaningful oncologic benefit when treated at a specialized center. As CRS ± HIPEC availability continues to expand, recognizing these care needs will help inform optimal care delivery for patients with PSM.

## Supplementary Information

Below is the link to the electronic supplementary material.

Supplementary file1 (DOCX 790 KB)

## Previous presentation

Data from this manuscript were presented as a plenary presentation at the Society for Surgery of the Alimentary Tract on 6 May 2025, in San Diego, CA.
