# Supplementary material for: Characteristics and Outcomes of External Referral Patients Undergoing Cytoreductive Surgery at an International Peritoneal Surface Malignancy Program
Source: Ann Surg Oncol. 2026 Jul 18;33(12):10195–204. doi: 10.1245/s10434-026-20115-3 (PMC13675356; doi:10.1245/s10434-026-20115-3)
Supplement: Supplementary file 1 [file 10434_2026_20115_MOESM1_ESM.docx]

**SUPPLEMENTARY APPENDIX**

**Supplementary Table 1:** Number of previous cytoreductions prior to the index operation at a quaternary peritoneal surface malignancy center.

|  |  | **In-System**  *(n=70)* | **Out-of-System**  *(n=101)* | **Total**  *(n=171)* | **p-value** |
| --- | --- | --- | --- | --- | --- |
| **Previous cytoreductions** | **Mean (SD)** | 0.69±0.97 | 0.70±0.95 | 0.70±0.96 | 0.976 |
|  | **Median (IQR)** | 0 (0-1) | 0 (0-1) | 0 (0-1) | 0.976 |
|  | **Categories** |  |  |  | 0.714 |
|  | 0 | 37 (56.9%) | 53 (53.5%) | 90 (54.9%) |  |
|  | 1 | 16 (24.6%) | 32 (32.3%) | 48 (29.3%) |  |
|  | 2 | 8 (12.3%) | 7 (7.1%) | 15 (9.1%) |  |
|  | 3 | 3 (4.6%) | 5 (5.1%) | 8 (4.9%) |  |
|  | 4 | 1 (1.5%) | 2 (2.0%) | 3 (1.8%) |  |

**Supplementary Table 2:** Distribution of referring physician prior to the index operation at a quaternary peritoneal surface malignancy center.

|  | **In-System**  *(n=70)* | **Out-of-System**  *(n=101)* | **p-value** |
| --- | --- | --- | --- |
| **Referring Physicians** | **N (%)** | **N (%)** | n/a |
| Surgical Oncologist | n/a | 61 (85.9%) |  |
| Gynecologic Oncologist | n/a | 6 (8.5%) |  |
| Medical Oncologist | n/a | 3 (4.2%) |  |
| General Surgeon | n/a | 1 (1.4%) |  |

*Only reported for OOS patients and subject to data available via care everywhere on EPIC.

**Supplementary Table 3:** Comparison of operative procedures between in-system and out-of-system patients who underwent cytoreductive surgery at a quaternary peritoneal surface malignancy center.

|  | **In-System**  *(n=70)* | **Out-of-System**  *(n=101)* | **Total**  *(n=171)* | **p-value** |
| --- | --- | --- | --- | --- |
| **Right diaphragm resection** | 15 (21.4%) | 22 (21.8%) | 37 (21.6%) | 0.956 |
| **Left diaphragm resection** | 7 (10.0%) | 22 (21.8%) | 29 (17.0%) | 0.044 |
| **Liver resection** | 19 (27.1%) | 28 (27.7%) | 47 (27.5%) | 0.933 |
| **Gall bladder resection** | 11 (15.7%) | 31 (30.7%) | 42 (24.6%) | 0.025 |
| **Spleen resection** | 15 (21.4%) | 29 (28.7%) | 44 (25.7%) | 0.284 |
| **Stomach Resection** | 6 (8.6%) | 17 (16.8%) | 23 (13.5%) | 0.120 |
| **Distal pancreas resection** | 7 (10.0%) | 10 (9.9%) | 17 (9.9%) | 0.983 |
| **Pancreaticoduodenectomy** | 1 (1.4%) | 0 (0.0%) | 1 (0.6%) | 0.228 |
| **Omental resection** | 32 (45.7%) | 46 (45.5%) | 78 (45.6%) | 0.983 |
| **Small bowel resection** | 28 (40.0%) | 65 (64.4%) | 93 (54.4%) | 0.002 |
| **Right colon resection** | 20 (28.6%) | 50 (49.5%) | 70 (40.9%) | 0.006 |
| **Left colon resection** | 11 (15.7%) | 17 (16.8%) | 28 (16.4%) | 0.846 |
| **Low anterior resection** | 17 (24.3%) | 42 (41.6%) | 59 (34.5%) | 0.019 |
| **Bladder resection** | 8 (11.4%) | 19 (18.8%) | 27 (15.8%) | 0.193 |
| **Ureter resection** | 32 (45.7%) | 53 (52.5%) | 85 (49.7%) | 0.385 |
| **Total abdominal hysterectomy** | 17 (24.3%) | 22 (21.8%) | 39 (22.8%) | 0.701 |
| **Bilateral salpingo-oopherectomy** | 20 (28.6%) | 21 (20.8%) | 41 (24.0%) | 0.241 |
| **Vaginal cuff resection** | 7 (10.0%) | 4 (4.0%) | 11 (6.4%) | 0.113 |
| **Abdominal wall resection** | 11 (15.7%) | 12 (11.9%) | 23 (13.5%) | 0.470 |
| **Ostomy creation** | 9 (12.9%) | 39 (38.6%) | 48 (28.1%) | <0.001 |

**Supplementary Table 4:** Comparison of heated intraperitoneal chemotherapy utilization and drugs among patients who underwent cytoreductive surgery at a quaternary peritoneal surface malignancy center.

|  | **IS** | **OOS** | **Total** | **p-value** |
| --- | --- | --- | --- | --- |
| **HIPEC** | **N (%)** | **N (%)** | **N (%)** |  |
| Yes | 22 (31.4%) | 74 (73.3%) | 96 (56.1%) | <0.001 |
| No | 48 (68.6%) | 27 (26.7%) | 75 (43.9%) |  |
| **HIPEC Drug** |  |  |  |  |
| Mitomycin C | 19 (27.1%) | 73 (73.0%) | 92 (54.1%) | <0.001 |
| Cisplatin | 3 (4.3%) | 1 (1.0%) | 4 (2.4%) | 0.164 |

**Supplementary Table 5:** Differences between clinic-demographic characteristics and outcomes between out-of-system patients based on time from referral to surgery at a quaternary peritoneal surface malignancy center.

|  | **1^st^ Quartile** | **2^nd^ Quartile** | **3^rd^ Quartile** | **4^th^ Quartile** | **p-value** |
| --- | --- | --- | --- | --- | --- |
| **N** | 21 (25.3%) | 21 (25.3%) | 21 (25.3%) | 20 (24.1%) |  |
| **Age^a^** | 51.1±10.3 | 56.5±11.3 | 56.8±12.4 | 52.7±11.3 | 0.283 |
| **Sex** |  |  |  |  |  |
| Male | 11 (52.4%) | 14 (66.7%) | 9 (42.9%) | 8 (40.0%) | 0.308 |
| Female | 10 (47.6%) | 7 (33.3%) | 12 (57.1%) | 12 (60.0%) |  |
| **Race** |  |  |  |  |  |
| White | 19 (90.5%) | 21 (100.0%) | 20 (95.2%) | 17 (85.0%) | 0.356 |
| Black | 0 (0.0%) | 0 (0.0%) | 0 (0.0%) | 1 (5.0%) |  |
| Asian | 1 (4.8%) | 0 (0.0%) | 0 (0.0%) | 2 (10.0%) |  |
| Hispanic | 0 (0.0%) | 0 (0.0%) | 1 (4.8%) | 0 (0.0%) |  |
| Other | 1 (4.8%) | 0 (0.0%) | 0 (0.0%) | 0 (0.0%) |  |
| **Histology** |  |  |  |  |  |
| LAMN/HAMN | 5 (23.8%) | 7 (33.3%) | 5 (23.8%) | 8 (40.0%) | 0.850 |
| Appendiceal adenocarcinoma |  |  |  |  |  |
| Well to moderately differentiated | 2 (9.5%) | 3 (14.3%) | 4 (19.0%) | 5 (25.0%) |  |
| Poorly differentiated | 4 (19.0%) | 5 (23.8%) | 7 (33.3%) | 3 (15.0%) |  |
| Colon adenocarcinoma |  |  |  |  |  |
| Well to moderately differentiated | 3 (14.3%) | 3 (14.3%) | 3 (14.3%) | 2 (10.0%) |  |
| Poorly differentiated | 3 (14.3%) | 2 (9.5%) | 1 (4.8%) | 2 (10.0%) |  |
| Ovarian carcinoma |  |  |  |  |  |
| High Grade Serous Ovarian | 1 (4.8%) | 0 (0.0%) | 0 (0.0%) | 0 (0.0%) |  |
| Borderline mucinous ovarian | 1 (4.8%) | 0 (0.0%) | 0 (0.0%) | 0 (0.0%) |  |
| Mesothelioma | 0 (0.0%) | 0 (0.0%) | 1 (4.8%) | 0 (0.0%) |  |
| Sarcoma | 1 (4.8%) | 0 (0.0%) | 0 (0.0%) | 0 (0.0%) |  |
| Other/Miscellaneous | 1 (4.8%) | 1 (4.8%) | 0 (0.0%) | 0 (0.0%) |  |
| **Prior Chemo** |  |  |  |  |  |
| No | 13 (61.9%) | 9 (42.9%) | 8 (38.1%) | 7 (35.0%) | 0.299 |
| Yes | 8 (38.1%) | 12 (57.1%) | 13 (61.9%) | 13 (65.0%) |  |
| **PCI^b^** | 12.0 (5.0-27.0) | 23.0 (15.0-31.0) | 18.0 (12.0-26.0) | 26.0 (14.0-35.0) | 0.248 |
| **PCI ranges** |  |  |  |  |  |
| <10 | 6 (37.5%) | 1 (5.6%) | 4 (20.0%) | 1 (6.7%) | 0.213 |
| 10-20 | 3 (18.8%) | 4 (22.2%) | 8 (40.0%) | 6 (40.0%) |  |
| 21-30 | 2 (12.5%) | 6 (33.3%) | 5 (25.0%) | 4 (26.7%) |  |
| >30 | 5 (31.2%) | 7 (38.9%) | 3 (15.0%) | 4 (26.7%) |  |
| **CC status** |  |  |  |  |  |
| CC0 | 16 (76.2%) | 9 (42.9%) | 12 (57.1%) | 8 (42.1%) | 0.181 |
| CC1 | 2 (9.5%) | 8 (38.1%) | 7 (33.3%) | 7 (36.8%) |  |
| CC2 | 0 (0.0%) | 2 (9.5%) | 1 (4.8%) | 0 (0.0%) |  |
| CC3 | 3 (14.3%) | 2 (9.5%) | 1 (4.8%) | 4 (21.1%) |  |
| **CC score at our facility** |  |  |  |  |  |
| CC0/1 | 18 (85.7%) | 17 (81.0%) | 19 (90.5%) | 15 (78.9%) | 0.749 |
| **Days between referral and surgery at our facility^b^** | 38.0 (32.0 50.0) | 92.0 (77.0 128.0) | 162.0 (153.0 172.0) | 242.5 (224.0 319.5) | <0.001 |
| **Estimated blood loss^b^** | 550.0 (250.0 850.0) | 1,200.0 (500.0-1,600.0) | 500.0 (300.0-1,000.0) | 750.0 (315.0-1,000.0) | 0.102 |
| **Operative time^b^** | 413.0 (351.0-616.0) | 599.0 (536.0-651.0) | 545.0 (381.0-645.0) | 556.5 (361.0-624.5) | 0.131 |
| **LOS^b^** | 9.0 (11.0-17.0) | 11.0 (12.0-18.0) | 8.0 (12.0-22.0) | 10.5 (18.5-23.0) | 0.844 |

LAMN: Low-grade appendiceal mucinous neoplasm, HAMN: High-grade appendiceal mucinous neoplasm, PCI: Peritoneal carcinomatosis index, CC: Completeness of cytoreduction, LOS: Lenth of stay, ^a^ Data reported as mean ± standard deviation, ^b^ Data reported as median and interquartile range

**Supplementary Figure 1:** Geographical distribution of out-of-system patients who underwent cytoreductive surgery at a quaternary peritoneal surface malignancy center.

**Supplementary Figure 2:** Kaplan-Meier survival analysis comparing the in-system and out-of-system cohorts overall and within the most common diagnosis subgroups, including LAMN/HAMN, appendiceal adenocarcinoma, and colorectal adenocarcinoma.
